# *Pseudodesulfovibrio cashew* sp. Nov., a Novel Deep-Sea Sulfate-Reducing Bacterium, Linking Heavy Metal Resistance and Sulfur Cycle

**DOI:** 10.3390/microorganisms9020429

**Published:** 2021-02-19

**Authors:** Rikuan Zheng, Shimei Wu, Chaomin Sun

**Affiliations:** 1 CAS Key Laboratory of Experimental Marine Biology & Center of Deep Sea Research, Institute of Oceanology, Chinese Academy of Sciences, Qingdao 266071, China; zhengrikuan15@mails.ucas.edu.cn; 2 Laboratory for Marine Biology and Biotechnology, Qingdao National Laboratory for Marine Science and Technology, Qingdao 266071, China; 3 College of Earth Science, University of Chinese Academy of Sciences, Beijing 100049, China; 4 Center of Ocean Mega-Science, Chinese Academy of Sciences, Qingdao 266071, China; 5 Department of Life Science, Qingdao University, Qingdao 266071, China; wushimei@qdio.ac.cn

**Keywords:** dissimilatory sulfate reduction, sulfur cycle, heavy metals, *dsr* genes, deep-sea, cold seep

## Abstract

Sulfur cycling is primarily driven by sulfate reduction mediated by sulfate-reducing bacteria (SRB) in marine sediments. The dissimilatory sulfate reduction drives the production of enormous quantities of reduced sulfide and thereby the formation of highly insoluble metal sulfides in marine sediments. Here, a novel sulfate-reducing bacterium designated *Pseudodesulfovibrio cashew* SRB007 was isolated and purified from the deep-sea cold seep and proposed to represent a novel species in the genus of *Pseudodesulfovibrio*. A detailed description of the phenotypic traits, phylogenetic status and central metabolisms of strain SRB007 allowed the reconstruction of the metabolic potential and lifestyle of a novel member of deep-sea SRB. Notably, *P. cashew* SRB007 showed a strong ability to resist and remove different heavy metal ions including Co^2+^, Ni^2+^, Cd^2+^ and Hg^2+^. The dissimilatory sulfate reduction was demonstrated to contribute to the prominent removal capability of *P. cashew* SRB007 against different heavy metals via the formation of insoluble metal sulfides.

## 1. Introduction

Sulfur is an essential element for life, which is widely found in the natural environment. The ocean represents a major reservoir of sulfur on Earth, with large quantities in the form of dissolved sulfate ion (SO_4_^2−^), which is the second most abundant anion next to chloride [1]. Marine sediments are the main sink for sea-water sulfate and the sedimentary sulfur cycle is a major component of the global sulfur cycle [2,3]. Remarkably, the sulfur cycle of marine sediments is primarily driven by dissimilatory sulfate reduction (DSR), which is mediated by sulfate-reducing bacterium (SRB) in many anaerobic environments [2]. SRB mediate two sulfate-reduction pathways: assimilatory sulfate reduction (ASR) and dissimilatory sulfate reduction (DSR). Ecologically, DSR plays a major role in the global sulfur and carbon cycles [4]. The canonical microbial pathway for dissimilatory sulfate reduction involves the initial reduction of sulfate (SO_4_^2−^) to sulfite (SO_3_^2−^) by a combination of sulfate adenylyltransferase (Sat) and adenylyl-sulfate reductase (AprAB), followed by reduction of SO_3_^2−^ to H_2_S (S^2−^) by a single enzyme (bisulfite reductase) [5]. The other pathway involves several enzymes (such as sulfite reductase, trithionate reductase and thiosulfate reductase) and intermediates (such as trithionates and thiosulfates) [6].

Given that the formation of a large amount of sulfide in the course of sulfate reduction and most of the heavy metals react with sulfide to form highly insoluble metal sulfides, SRB-mediated dissimilating sulfate reduction is proposed an effective way to cope with the stress of many harmful metal ions which broadly distribute in marine sediments [7,8]. Meanwhile, an accepted clever idea was developed by researchers to remove heavy metals from the environment by utilizing dissimilating sulfate reduction mediated by different SRB [9,10]. Hence, SRB-mediated metal sulfide precipitation represents a potentially useful means of bioremediation of metal ion contaminants, which is an attractive alternative over physicochemical methods [9,11].

Overall, rather than being a simple cycle, composed of anaerobic bacterial reduction of sulfate to hydrogen sulfide and aerobic reoxidation of H_2_S to SO_4_^2^^−^, the transformations of sulfur in marine sediments also links to the cycles of carbon, nitrogen, iron, manganese and other important elements [12,13,14]. Therefore, it is of utmost importance to identify novel SRB mediating the sulfur cycle in marine sediments, which will be of great advantage to disclose novel mechanisms and develop more powerful bioremediation products. *Pseudodesulfovibrio* is a new genus of SRB, which was originally proposed and reclassified from four species of the genus *Desulfovibrio* by Cao et al. in 2016 [15]. To date, most species of the genus *Pseudodesulfovibrio* have been isolated from marine sediments, including *Pseudodesulfovibrio profundus* [16], *Pseudodesulfovibrio portus* [17], *Pseudodesulfovibrio piezophilus* [18] and *Pseudodesulfovibrio indicus* [15], which may indicate that these strains play important roles in driving the sulfur cycle in marine sediments. However, to date, no results about the sulfur cycle and heavy metal resistance mediated by *Pseudodesulfovibrio* other than taxonomic data have been published.

In the present study, a novel species of the genus *Pseudodesulfovibrio*, SRB007, was isolated from the deep-sea cold seep and proposed as the type strain for this novel species. Furthermore, the taxonomy and typical physiological properties closely related to sulfur cycle and heavy metal resistance were disclosed through the combination of genomic and biochemical methods, providing a clue to understanding the coupling of different elements in the deep-sea cold seep and a potential candidate for developing bioremediation products in the future.

## 2. Materials and Methods

### 2.1. Bacterial Strains and Culture Conditions

The samples were collected by *RV KEXUE* from the cold seep in the South China Sea (E 119°17′07.322″, N 22°06′58.598″) as described previously [3,19]. The sediment samples were cultured at 28 °C for one month in an anaerobic enrichment medium containing (per litre of seawater): 1 g NH_4_Cl, 1 g NaHCO_3_, 1 g CH_3_COONa, 0.5 g KH_2_PO_4_, 0.2 g MgSO_4_·7H_2_O, 1 g peptone, 1 g yeast extract, 0.7 g cysteine hydrochloride, 1 mL 0.1% (*w/v*) resazurin (the pH was adjusted to 7.0) and the medium was prepared anaerobically as previously described [20]. The cultures were purified by repeated use of the Hungate roll-tube method. Single colonies were picked by sterilized bamboo skewers and then cultured in the same medium. The process of isolation was repeated several times until the isolates were deemed to be axenic. The purity of the isolate was confirmed routinely by transmission electron microscopy (TEM) and by repeated partial sequencing of the 16S rRNA gene. Then the single colony was transferred to a new medium (D195c) containing (per litre of seawater): 1.0 g yeast extract, 2.0 g peptone, 2.2 g sodium lactate, 2.0 g Na_2_SO_4_, 3.3 g PIPES (Piperazine-1,4-bisethanesulfonic acid), 1.0 g cysteine hydrochloride, 1 mL 0.1% (*w/v*) resazurin; the pH of the medium was adjusted to 7.0 with NaOH. The cultures were maintained at 30 °C in an anaerobic chamber (Longyue, China) under a gas mixture of 90% N_2_, 5% CO_2_ and 5% H_2_.

### 2.2. Transmission Electron Microscopy (TEM) Observation

To observe the morphological characteristics of *P. cashew* SRB007, the cells were examined using TEM with a JEOL JEM 12000 EX (equipped with a field emission gun) at 100 kV. The cell suspension of *P. cashew* SRB007 was washed with Milli-Q water and centrifuged at 4000× *g* for 5 min. Subsequently, the sample was taken by immersing copper grids coated with a carbon film for 20 min in the bacterial suspensions and washed for 5 min in distilled water and dried for three hours at room temperature [21].

### 2.3. Genomic Sequencing and Analysis

To obtain the whole genome of *P. cashew* SRB007, the total chromosomal DNA of this bacterium was extracted. The DNA library was prepared, using the Ligation Sequencing Kit (SQK-LSK109) and sequenced, using a FLO-MIN106 vR9.4 flow-cell for 48 h on MinKNOWN software v1.4.2 (Oxford Nanopore Technologies (ONT), Kidlington, UK). Whole-genome sequence determination was carried out with the Oxford Nanopore MinION (Oxford, Kidlington, UK, United Kingdom) and Illumina MiSeq sequencing platform (San Diego, CA, USA) and a hybrid approach was further utilized for genome assembly using reads from both platforms. Base-calling was performed via Albacore software v2.1.10 (Oxford Nanopore Technologies). Nanopore reads were processed with the Poretools toolkit for the purposes of quality control and downstream analysis [22] and the filtered reads were assembled by Canu v1.8 [23]. The whole genome was finally assembled into a single contig and was manually circularized by deleting an overlapping end. Based on the whole genome of *P. cashew* SRB007, full-length 16S rRNA, *dsrABCDE* and other genes related to sulfate reduction were obtained and applied to different analyses. There are three 16rRNA gene copies in the genome. We selected one 16S rRNA gene (National Coalition Building Institute (NCBI) GenBank accession number: AF418172) for analysis in this study.

### 2.4. Phylogenetic Analysis

Sequences of 16S rRNA and *dsrAB* of *P. cashew* SRB007 and other related taxa used for phylogenetic analysis were all obtained from the NCBI GenBank. Phylogenetic analysis was performed using the software MEGA version 6.0 [24]. The phylogenetic tree was constructed by the neighbor-joining algorithm [25], maximum likelihood [26] and minimum-evolution methods [27]. The numbers above or below the branches were bootstrap values based on 1000 replicates.

### 2.5. Physiological and Chemotaxonomic Assays of P. cashew SRB007

Morphological characteristics and purity of *P. cashew* SRB007 were observed by TEM. Growth assays were performed at atmospheric pressure, using Hungate tubes containing basal medium (3.3 g PIPES, 1.0 g cysteine hydrochloride, 1 mL 0.1% (*w/v*) resazurin; the pH of the medium was adjusted to 7.0 with NaOH) and different electron donors at 20 mM (fumarate, formate, pyruvate, lactate, malate, methanol, fructose, propionate, butyrate, succinate, glycine, ethanol). Elemental sulfur (1%, *w/v*), sulfate (20 mM), sulfite (20 mM), thiosulfate (20 mM), nitrate (5 mM) and nitrite (10 mM) were tested as terminal electron acceptors. The temperature, pH and NaCl concentration ranges for the growth of *P. cashew* SRB007 were determined in duplicate experiments using basal medium supplemented with lactate (20 mM) as electron donor and sulfate (20 mM) as previously described [28]. Temperatures for growth were tested between 4 and 80 °C. The pH range for growth was tested from pH 3.0 to pH 11.0 (at 30 °C) with increments of 0.5 pH units. Salt resistance was determined by directly weighing NaCl (0–100 g L^−1^) into the Hungate tubes before packaging the basal medium. For each condition, three biological replicates were performed.

### 2.6. Heavy Metal Removal Assay and Qualitative Energy-Dispersive Spectrometry (EDS) Analysis

To determine the Hg^2+^ removal rate of *P. cashew* SRB007 as an example, *P. cashew* SRB007 was incubated at 30 °C in modified D195c anaerobic medium supplemented with 0.1 mM HgCl_2_. The samples were collected at 1 d, 2 d, 3 d and 4 d, respectively. The supernatant of the culture was collected by centrifugation (12,000× *g*, 5 min). After this, the supernatant was thoroughly digested with perchloric acid and nitric acid and diluted with Milli-Q water for Hg^2+^ concentration detection. The final concentration of dissolved Hg^2+^ was measured with an inductively coupled plasma source mass spectrometer (Optima 7300 DV, PerkinElmer). The removal rate was calculated as (original concentration—final concentration)/ original concentration × 100%. The determination of the removal rate of other heavy metal ions (Co^2+^, Ni^2+^ and Cd^2+^) against *P. cashew* SRB007 was performed as mentioned above. In addition, the precipitation was washed with Milli-Q water three times and then ultrasonic decomposition was performed for 30 min. The sample was collected by centrifugation (12,000× *g*, 10 min) and washed with Milli-Q water for three times. Finally, the sample was dried in an oven for 3 h at 80 °C and then thin-coated by Au for EDS analysis using a model 550i (IXRF SYSTEMS, Austin, TX, USA).

### 2.7. RNA Extraction, Reverse Transcription and Quantitative Real-Time Polymerase Chain Reaction (qRT-PCR)

For quantitative real-time polymerase chain reaction (qRT-PCR), cells of *P. cashew* SRB007 challenged with different heavy metal ions (2.5 mM Co^2+^, 2.5 mM Ni^2+^, 2.0 mM Cd^2+^ and 0.1 mM Hg^2+^) were grown in modified D195c anaerobic medium at 30 °C for 2 days, then 2 mL of these cells were harvested and centrifuged at 12,000× *g* for 5 min, respectively. Total RNAs were extracted using the Trizol reagent (Solarbio, Beijing, China) and the RNA concentration was measured using Qubit® RNA Assay Kit in Qubit^®^ 2.0 Fluorometer (Life Technologies, CA, USA). Then RNA was reverse transcribed into cDNA and the transcriptional levels of different genes were determined by qRT-PCR using SybrGreen Premix Low rox (MDbio, shenzen, China) and the QuantStudio^TM^ 6 Flex (Thermo Fisher Scientific, Waltham, MA, USA). We used 16S rRNA as an internal reference and the purpose gene expression was calculated using the 2^−ΔΔCt^ method, with each transcript signal normalized to 16S rRNA [29]. Transcript signals for each treatment were compared to the transcript signals of the control group. The transcription levels of each *dsr* gene were normalized to the 16S rRNA gene. Specific primers for *dsrA*, *dsrB*, *dsrD*, *dsrN*, *dsrC*, *dsrE* and 16S rRNA of *P. cashew* SRB007 were designed using Primer 5.0 (Supplementary Table S1). All qRT-PCR runs were performed in three biological and three technical replicates.

### 2.8. Data Deposit 

The complete genome sequence of *P. cashew* SRB007 has been deposited at GenBank under the accession number CP046400. The NCBI GenBank accession number for the 16S rRNA gene sequence of *P. cashew* SRB007 is AF418172.

## 3. Results

### 3.1. Isolation and Identification of a Novel Deep-Sea Sulfate-Reducing Bacterium P. cashew SRB007 

**Figure 1:**
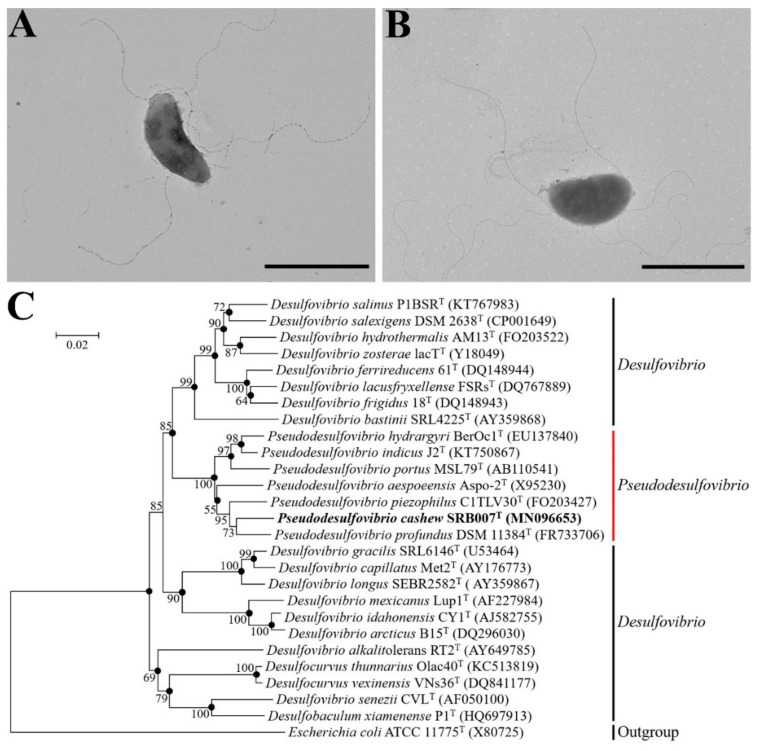
Morphology observation and phylogenetic analysis of *P. cashew* SRB007. (**A**,**B**) Morphology observation of *P. cashew* SRB007 by transmission electron microscopy (TEM). The bars in panels A and B were both 2 μm. (**C**) Phylogenetic tree based on 16S rRNA gene sequences of *P. cashew* SRB007 and related strains reconstructed by the neighbor-joining method. GenBank accession numbers are given in parentheses after strain names. Bootstrap values were based on 1000 replicates. Bar, 0.02 substitution per nucleotide position. The strain indicated with bold font represented strain SRB007 identified in this study. *Escherichia coli* ATCC 11775^T^ (GenBank accession number X80725) was used as the outgroup.

During isolation of uncultured microorganisms from the deep-sea cold seep, a potential novel species (strain SRB007) of SRB was obtained after several rounds of purification, which showed 97.34% similarity of 16S rRNA sequence to that of *Pseudodesulfovibrio profundus* DSM 11384^T^ (also named 500–1), the type strain of the genus *Pseudodesulfovibrio*, isolated from a deep sediment layer in the Japan Sea [16]. Strain SRB007 was mesophilic, strictly anaerobic and Gram-stain-negative, while spores were never observed. Under TEM observation, the cells of strain SRB007 were cashew-shaped, approximately 1.0–2.5 × 0.3–0.7 µm in size and had peritrichous flagella (Figure 1A,B). To further identify the taxonomic status of strain SRB007, we performed the phylogenetic analyses with 16S rRNA genes from some cultured representatives of the family Desulfovibrionaceae. All of the phylogenetic trees of 16S rRNA showed that strain SRB007 fell within the cluster comprising *Pseudodesulfovibrio* species and the closest species in the NCBI database was *Pseudodesulfovibrio profundus* DSM 11384^T^ (97.34% sequence similarity) [16], and the next more closely related recognized species were *Pseudodesulfovibrio piezophilus* C1TLV30^T^ (96.68% similarity) [18] and *Pseudodesulfovibrio indicus* J2^T^ (95.71% similarity) (Figure 1C, Figures S1 and S2). These results allowed us to propose the strain SRB007 as a representative of a novel species belonging to the *Pseudodesulfovibrio* genus. Thus, the strain SRB007 was proposed as the type strain and designated as *Pseudodesulfovibrio cashew* SRB007^T^.

### 3.2. Physiological and Chemotaxonomic Characteristics of P. cashew SRB007

To further gain insights into the lifestyle of *P. cashew* SRB007, a series of physiological and chemotaxonomic characteristics of this bacterium were investigated. *P. cashew* SRB007 had a high survival ability to tolerate different salt concentrations (0–100 g/L NaCl) (Table 1), which was according well with other typical SRB isolated from the ocean [18,30,31,32,33,34,35] (Supplementary Table S2), and this property might benefit this bacterium to survive in various hypersaline habitats. Compared with the closely related type strain *P. profundus* DSM 11384^T^ and other SRB, *P. cashew* SRB007 showed a wider range to utilize different substrates as electron donors (including fumarate, malate, succinate, formate, lactate, methanol and ethanol) and acceptors (including sulfate, sulfite, thiosulfate, nitrate and nitrite) (Table 1), which endowed *P. cashew* SRB007 with a strong flexibility in different environments. Consistently, *P. cashew* SRB007 contained more genes involved in sulfur and nitrogen metabolisms than strain 11384^T^, and these genes included *dsrD*, *dsrC*, *dsrE*, *dsrP*, *dsrM* for sulfur metabolism and *nifS*, *nifJ*, *nifN* for nitrogen metabolism.

The major polar lipids in *P. cashew* SRB007 were phosphatidylethanolamine, diphosphatidylglycerol, phosphatidylglycerol, unidentified glycolipid and unknown aminoglycolipids (Figure S3). The predominant fatty acids (>10%) were iso- C_15:0_, C_16:0_ and iso- C_17:0_ (Table 1). The amount of iso- C_15:0_, C_16:0_ and iso- C_17:0_ in *P. cashew* SRB007 (38.87%, 21.28% and 11.10%) were higher than those found in *P. profundus* DSM 11384^T^ (22.61%, 7.50% and 1.15%, respectively), while the amount of anteiso- C_15:0_ in *P. cashew* SRB007 (8.80%) was lower than that found in *P. profundus* DSM 11384^T^ (15.22%) (Supplementary Table S3). The distinctive composition of fatty acids of *P. cashew* SRB007 facilitates its better adaptation to atmospheric pressure as a typical deep-sea bacterium, as described previously [36].

### 3.3. Description of Pseudodesulfovibrio Cashew sp. Nov. 

*Pseudodesulfovibrio cashew* (ca’sh.ew N.L. n. cashew nut, referring to its similar appearance to a cashew nut).

Cells of strain SRB007^T^ are Gram-stain-negative, strictly anaerobic, cashew-shaped, 1.0–2.5 µm in length and 0.3–0.7 µm in width, motile by peritrichous flagella. The temperature range for growth is 16–45 °C with an optimum at 30 °C. Growing at pH values of 5.5–8.5 (optimum, pH 7.0) and at NaCl of 0–100 g/L (optimum, 50 g/L). Fumarate, methanol, ethanol, formate, lactate, succinate and malate are oxidized with sulfate reduction. Sulfate, sulfite, thiosulfate, nitrate and nitrite serve as electron acceptors. The major polar lipids are phosphatidylethanolamine, diphosphatidylglycerol, phosphatidylglycerol, unidentified glycolipid and unknown aminoglycolipids. It contains significant proportions (>10%) of the cellular fatty iso-C_15:0_, C_16:0_, iso-C_17:0_.

The type strain, SRB007^T^ (=KCTC 15990^T^ =MCCC 1K04423^T^), was isolated from deep-sea sediments of cold seep, P.R. China. The DNA G+C content of the type strain is 59.94%.

### 3.4. Dissimilatory Sulfate Reduction-Related Genes Existing in the Genome of P. cashew SRB007

To gain more insights into sulfate reduction-related characteristics of *P. cashew* SRB007, its whole genome was sequenced (Figure S4). The genome size of *P. cashew* SRB007 was 3,909,950 bp with a DNA G+C content of 59.94%. The number of contig was 1, the total of N50 was 3,909,950 and the sequencing depth was 50.0×. Annotation of the genome of *P. cashew* SRB007 consisted of 3499 coding sequences that included 68 RNA genes (9 rRNA genes, 55 tRNA genes and 4 other ncRNAs).

**Figure 2:**
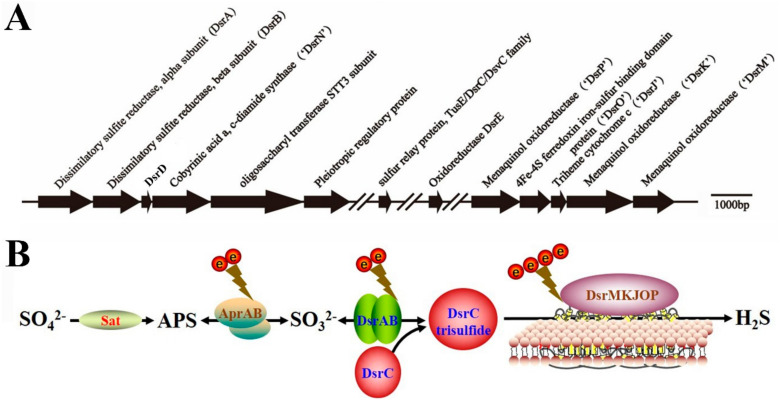
Genomic analysis of dissimilatory sulfate reduction of *P. cashew* SRB007. (**A**) The gene cluster containing the typical dissimilatory sulfite reductase operon and associated genes identified in the genome of *P. cashew* SRB007. (**B**) Proposed pathway of dissimilatory sulfate reduction of *P. cashew* SRB007. Sat, sulfate adenylyltransferase; APS, adenylyl sulfate; AprAB, adenylylsulfate reductase; DsrABC, reverse-type dissimilatory sulfite reductase; DsrMKJOP, sulfite reduction-associated complex.

Notably, *P. cashew* SRB007 contained a complete gene cluster composed of genes encoding proteins involved in dissimilatory sulfate reduction (Figure 2A). Among them, *dsrAB*, *dsrC* together with *dsrMKJOP* encode all the necessary components of the Dsr complex required for sulfate reduction [37]. In this complex, DsrA and DsrB are annotated as different subunits of sulfite reductase; DsrC is annotated as sulfur relay protein; DsrK, DsrM and DsrP are annotated as menaquinone oxidoreductase; DsrJ is annotated as triheme cytochrome C; DsrO is annotated as 4Fe-4S ferredoxin iron-sulfur binding domain protein. The other three genes (*dsrD*, *dsrE* and *dsrN*) which are commonly present in *dsr* operons and encode proteins not directly involved in electron shuttling during sulfate reduction, were also identified in the genome of *P. cashew* SRB007. In combination with the discovery of other genes involved the sulfate reduction in the genome of *P. cashew* SRB007, an inferred pathway of dissimilatory sulfate reduction mediating reduction of sulfate to sulfide is shown in Figure 2B. In this pathway, SO_4_^2-^ was reduced to SO_3_^2-^ by a combination of Sat and AprAB; then SO_3_^2-^ was reduced to DsrC trisulfide by DsrAB and DsrC; the DsrC trisulfide produced was finally reduced to sulfide by the DsrMKJOP complex.

### 3.5. Dissimilatory Sulfate Reduction-Related Genes Contribute to the Prominent Capability of P. cashew SRB007 against Different Heavy Metals

**Figure 3:**
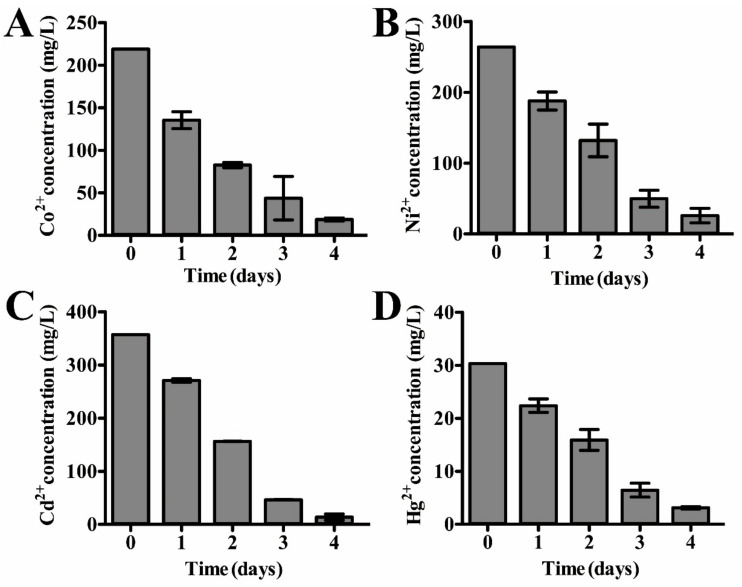
Measurement of the removal efficiency of heavy metals by *P. cashew* SRB007. The concentrations of Co^2+^ (**A**), Ni^2+^ (**B**), Cd^2+^ (**C**) and Hg^2+^ (**D**)were measured at 0, 1, 2, 3 and 4 d, respectively. The removal efficiency was calculated as described in the method part.

**Figure 4:**
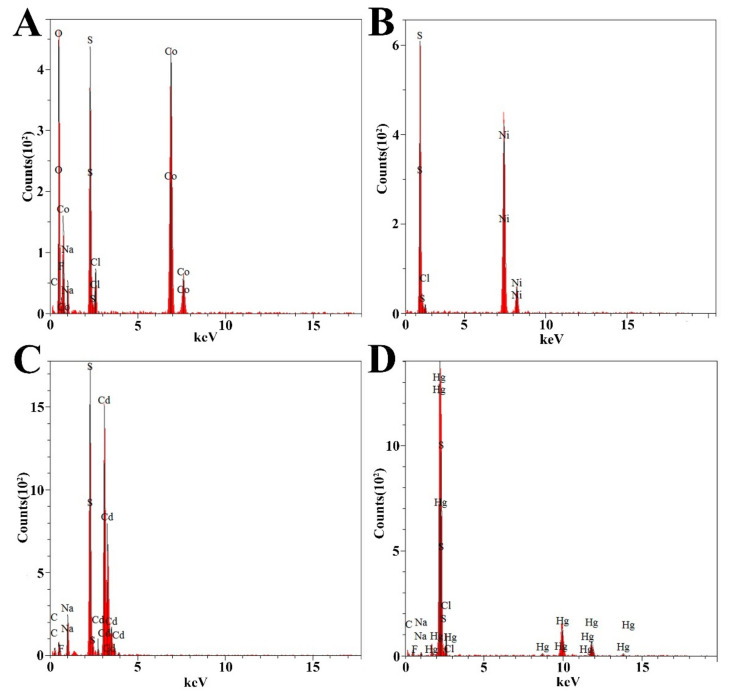
Energy-dispersive spectrometry spectra of the precipitates formed by *P. cashew* SRB007 when cultured in the medium containing different concentrations of Co^2+^ (**A**), Ni^2+^ (**B**), Cd^2+^ (**C**) and Hg^2+^ (**D**), respectively.

Given that *P. cashew* SRB007 is a typical sulfate-reducing bacterium, we next sought to explore its resistance to heavy metals and evaluate its potential in the field of bioremediation as previously described [9,10]. With this, the growth status of *P. cashew* SRB007 was checked when challenged with different heavy metals including Co^2+^, Ni^2+^, Cd^2+^ and Hg^2+^, which are common metal ions existing in the deep-sea and industrial waste water. The results showed that *P. cashew* SRB007 had an MIC (minimum inhibitory concentration) of 2.5 mM, 2.5 mM, 2.0 mM and 0.10 mM toward Co^2+^, Ni^2+^, Cd^2+^ and Hg^2+^, respectively. Furthermore, the removal capabilities of *P. cashew* SRB007 toward the above tested heavy metals were checked. The results showed that with the extension of culturing time, the removal rate of heavy metals gradually increased and finally stabilized at 91.5%, 90.2%, 96.2% and 89.8% toward Co^2+^, Ni^2+^, Cd^2+^ and Hg^2+^, respectively, at the end of the fourth day (Figure 3). It is worth noting that there were obvious precipitates formed in the bottom of the medium when *P. cashew* SRB007 was cultured with different metals, and the amount of precipitates increased with the length of incubation time. Given that *P. cashew* SRB007 is a typical SRB and has the potential to form S^2-^, we propose that these precipitates were metal sulfides. Indeed, these precipitates were further demonstrated to be CoS, NiS, CdS and HgS by energy-dispersive spectrometry (EDS) analyses (Figure 4), strongly indicating that dissimilatory sulfate reduction contributes to the resistance and removal capabilities of *P. cashew* SRB007 against different heavy metals. Taken together, *P. cashew* SRB007 had a prominent removal rate toward different heavy metals (Co^2+^, Ni^2+^, Cd^2+^ and Hg^2+^) by forming insoluble metal sulfides, indicating *P. cashew* SRB007 might be applied to the treatment of sewage to remove heavy metals in the future.

**Figure 5:**
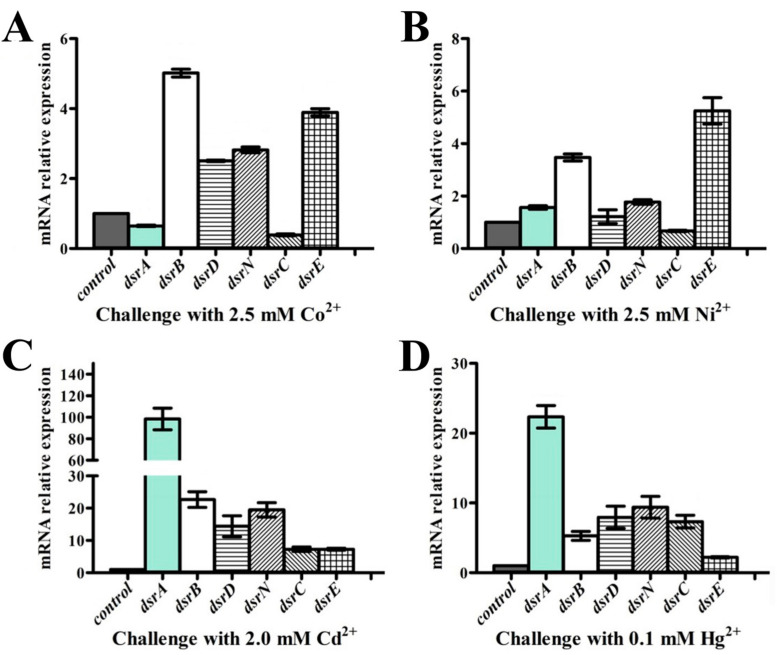
Quantitative real-time polymerase chain reaction (qRT-PCR) analysis of the expression of genes within *dsr*-operon of *P. cashew* SRB007 challenged with 2.5 mM Co^2+^ (**A**), 2.5 mM Ni^2+^ (**B**), 2.0 mM Cd^2+^ (**C**) and 0.1 mM Hg^2+^ (**D**), respectively.

To confirm the participation of the dissimilatory sulfate reduction associated genes in the course of coping with different heavy metals by *P. cashew* SRB007, the expressions of several important genes within the gene cluster mentioned in Figure 2A were checked by real-time quantitative PCR. Clearly, the expression of *dsrABDNCE* was significantly up-regulated from ~6- to ~100- fold when challenged with Cd^2+^ and Hg^2+^. However, the expression of *dsrABE* was only up-regulated from ~2- to ~5- fold when challenged with Co^2+^ and Ni^2+^ (Figure 5). Given that Cd^2+^ and Hg^2+^ are high toxicity metal ions and Co^2+^ and Ni^2+^ are essential elements to life, it is reasonable to see the results that the expression of *dsrABDNCE* was much higher when challenged with Cd^2+^ and Hg^2+^ compared with other low-toxicity metal ions like Co^2+^ and Ni^2+^.

### 3.6. Proposed Lifestyle of P. cashew SRB007

**Figure 6:**
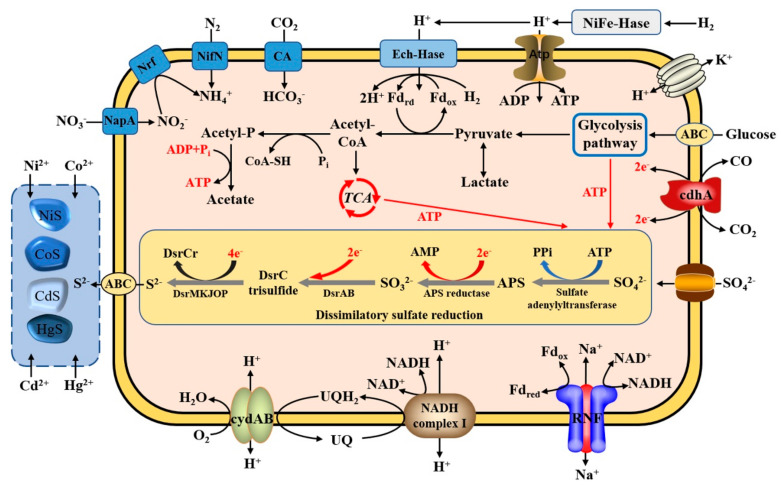
Proposed lifestyle of *P. cashew* SRB007. Abbreviations: TCA, tricarboxylic acid cycle; FeFe-Hase, FeFe-hydrogenase; Atp, ATP synthase; ATP, 5’-Adenylate triphosphate; ADP, adenosine diphosphate; AMP, adenosine monophosphate; Ech-Hase, energy-conserving membrane-bound hydrogenase; CA, carbonic anhydrase; NifN, nitrogenase; Nrf, cytochrome c nitrite reductase; NapA, nitrate reductase catalytic subunit; cydAB, cytochrome bd-I ubiquinol oxidase subunit; cdhA, CO dehydrogenase/acetyl-CoA synthase complex subunit epsilon; UQ, ubiquinone; UQH_2_, Cytochrome C reductase; RNF, the sodium translocating NADH: Ferredoxin oxidoreductase; DsrAB, dissimilatory sulfite reductases; PPi, pyrophosphoric acid.

In combination of the genomic and physiological traits of *P. cashew* SRB007, a proposed lifestyle is shown in Figure 6. First, there were corresponding genes encoding proteins involved in dissimilatory sulfate reduction in the genome of *P. cashew* SRB007, which could reduce SO_4_^2-^ to S^2−^ and then be transported outside the cell to bind to surrounding heavy metals to form metal sulfide precipitates in response to stress from the outside (Figure 6). In addition, the genes in charge of sugar transport and the glycolysis pathway that generate much energy were also discovered in the bacterial genome (Figure 6). Moreover, nearly all of the genes involved in the nitrogen fixation process including *nifHDK* encoding molybdenum-iron nitrogenase, *nifBENSU* encoding nitrogen-fixing assembly proteins, and *nifA* encoding transcriptional regulator proteins, were found in the genome of *P. cashew* SRB007 (Supplementary Table S4) [38]. There were also some genes encoding the complete components of NiFe hydrogenase, Ech hydrogenase and anaerobic carbon-monoxide dehydrogenase that catalyzed the oxidation of carbon monoxide to carbon dioxide or the reverse reaction. Finally, *P. cashew* SRB007 also possesses the sodium translocating NADH (Nicotinamide adenine dinucleotide) ferredoxin oxidoreductase (RNF complex), the proton translocating NADH quinone oxidoreductase (complex I), and cytochrome *bd* ubiquinol oxidase (cydAB). Both the complex I and cytochrome *bd* ubiquinol oxidase interact with the menaquinone pool, which can form a simple electron transport chain to generate energy [39]. All the above results indicate that *P. cashew* SRB007 could generate energy via multiple pathways, which provides enough energy for bacterial sulfur cycle and heavy metal tolerance.

## 4. Discussion

Sulfur is a key element in nature, whose transformation and status are critically dependent upon microbial activities. The sulfur cycle in marine sediments is primarily driven by dissimilatory sulfate reduction to sulfide by anaerobic SRB [1]. There have been extensive studies of sedimentary sulfur cycle across the global ocean and focus on geological microscopic transformations of the two end-members sulfate and sulfide [40]. As previously reported, the estimates of quantities in the marine sediments of deep-sea suggest that SRB account for approximately 5–25% of the microbial biomass in the surface of sulfate-rich zones and may reach up to approximately 30–35% in the sulfate-methane transition zone [41]. Given that the deep-sea cold seep is a very special environment where is also abundant methane and sulfate, it may be one of the best locations to study the sulfur cycle mediated by SRB. However, because of the difficulty of sample collection and absence of pure SRB cultures from the deep-sea cold seep, it is of utmost importance to obtain typical SRB to explore the unknown mechanisms about the sulfur cycle occurring in this special environment.

In the present study, a novel sulfate-reducing bacterium designated *P. cashew* SRB007 was isolated and purified from the deep-sea cold seep and proposed to represent a novel species in the genus of *Pseudodesulfovibrio* (Figure 1). To date, only five species were reported in the genus of *Pseudodesulfovibrio* and four of them were isolated from marine sediments, including *P. profundus* [16], *P. portus* [17], *P. piezophilus* [18] and *P. indicus* [15]. *P. cashew* SRB007 reported in this study is the first species isolated from the deep-sea cold seep of the genus of *Pseudodesulfovibrio. P. cashew* SRB007 can grow well under the conditions of 16 to 45 °C, 0–100 g/L NaCl and utilize fumarate, methanol, ethanol, formate, lactate, succinate, malate as electron donors and sulfate, sulfite, thiosulfate, nitrate, nitrite as electron acceptors (Table 1). Thus, it can be seen that *P. cashew* SRB007 possesses a wide growth condition and a broad range of substrates involved in sulfur and nitrogen cycles.

Given the isolation location of *P. cashew* SRB007, it is reasonable to speculate that this bacterium should have a strong capability to resist different heavy metals. As respected, *P. cashew* SRB007 could resist and remove high concentrations of Co^2+^, Ni^2+^, Cd^2+^ and Hg^2+^ (Figure 3). Previous research has reported that heavy metal removal and sulfate reduction correlated with each other [42]. As *P. cashew* SRB007 is a sulfate-reducing bacterium, it is logical to infer that this bacterium removes heavy metals through sulfate reduction to form metal sulfide precipitation (Figure 4). Indeed, the expression of the dissimilatory sulfite reduction genes (*dsrABCDEMNKJOP*) of *P. cashew* SRB007 was demonstrated to correlate with its heavy metals resistance and removal capabilities (Figure 2 and Figure 5). Overall, *P. cashew* SRB007 is a novel deep-sea sulfate-reduction bacterium that grows well in atmospheric pressure and possesses a strong capability for removing many harmful heavy metals.

It is worth noting that many metal-polluted wastewaters, such as acid mine wastewater and industrial metallurgic wastewater, contain high concentrations of sulfate and toxic heavy metals, which are similar to that of the deep sea and pose a serious threat to the environment when discharged untreated [43,44]. Based on the understanding of the detoxification mechanism used by SRB to reduce the toxicity of heavy metals, it is possible to develop an efficient and environment friendly metal repair technology. Undoubtedly, *P. cashew* SRB007 is a good candidate to develop the corresponding bioremediation product as shown in Figure S5 in the future, given its strong environmental adaptive and heavy metal-resistant capabilities.

Furthermore, the sulfur cycle is tightly interwoven with other important element cycles (carbon, nitrogen, manganese and iron) in marine sediments [2]. To date, most of the mechanisms of the sulfur cycle and its coupling with other elements mediated by SRB have been uncovered. Given that *P. cashew* SRB007 is a typical deep-sea cold seep sulfate-reducing bacterium and could use many forms of organic and inorganic matter as energy sources (Figure 6), it will be a great interest to explore in depth the mechanisms of the sulfur cycle linking with other elements of this bacterium in the future.

## 5. Conclusions

Here, a novel sulfate-reducing bacterium *Pseudodesulfovibrio cashew* SRB007 was isolated from a deep-sea cold seep and proposed as the type strain for a novel species. The taxonomy and typical physiological properties closely related to the sulfur cycle, heavy metal resistance and their co-relationship were disclosed through a combination of genomic and biochemical methods. Given the absence of pure cultures of typical SRB isolated from the deep-sea cold seep, our work provides a good model for studying the sulfur cycle which can be coupled with other elements and is a potential candidate to develop bioremediation products in the future.

## Figures and Tables

**Table 1:** Differential physiological characteristics of *P. cashew* SRB007^T^ and the closely related type strain *P. profundus* DSM 11384^T^. Strains: 1, *P. cashew* SRB007^T^ (all data from this study); 2, *P. profundus* DSM 11384^T^ (all data from this study except DNA G+C content and polar lipids). **+**, Positive result or growth; −, negative result or no growth; NR, not reported.

Characteristic	1	2
Temperature range		
for growth (°C)	16–45	20–45
Optimum	30	28
pH range for growth	5.5–8.5	6.0–8.0
Optimum	7.0	7.0
NaCl range for growth (%)	0–10.0	2.0–10.0
Optimum	5.0	6.0
Electron donors		
Fumarate	+	−
Malate	+	+
Lactate	+	+
Methanol	+	−
Ethanol	+	−
Formate	+	−
Succinate	+	−
Electron acceptors		
Sulfate	+	+
Sulfite	+	−
Thiosulfate	+	−
Nitrate	+	−
Nitrite	+	−
Polar lipids Major fatty acids (>10%) DNA G+C content (%)	phosphatidylethanolamine (PE), diphosphatidylglycerol (DPG), phosphatidylglycerol (PG), unidentified glycolipid (GL), unknown aminoglycolipid (AGL) iso-C_15:0_, C_16:0_, iso-C_17:0_ 59.94	NR iso-C_15:0_, anteiso-C_15:0_ NR
Isolation source	Deep-sea sediments	Marine sediments

## Data Availability

The data presented in this study are openly available in NCBI GenBank, reference numbers (CP046400 and AF418172) for the genome and 16S rRNA gene sequence of *P. cashew* SRB007, respectively.

## Supplementary Materials

The following are available online at https://www.mdpi.com/2076-2607/9/2/429/s1, Figure S1: Maximum-likelihood tree based on 16S rRNA gene sequences of P. cashew SRB007 and related taxa. Figure S2: Minimum-evolution tree based on 16S rRNA gene sequences of P. cashew SRB007 and related taxa. Figure S3: The polar lipids of strain P. cashew SRB007 as revealed by two-dimensional TLC. Figure S4: Circular diagram of the genome of P. cashew SRB007. Figure S5: A proposed model of heavy metal removal equipment mediated by P. cashew SRB007. Table S1: Primers used for qRT-PCR in this study. Table S2: Summary of sulfate reducer species and physiological characteristics of some marine SRB. Table S3: Comparison of the main fatty acids (%) of P. cashew SRB007T with its closest relative P. profundus DSM 11384T. Table S4. Hypothetical products of functional genes in the genome of *P. cashew* SRB007 that were used to reconstruct the metabolic network.
